# The epidemiology of mental and substance use disorders in Australia 2020–22: Prevalence, socio-demographic correlates, severity, impairment and changes over time

**DOI:** 10.1177/00048674241275892

**Published:** 2024-09-08

**Authors:** Tim Slade, Joshua Vescovi, Cath Chapman, Maree Teesson, Vikas Arya, Jane Pirkis, Meredith G Harris, Philip M Burgess, Damian Santomauro, Siobhan O’Dean, Caley Tapp, Matthew Sunderland

**Affiliations:** 1 The Matilda Centre for Research in Mental Health and Substance Use, The University of Sydney, Sydney, NSW, Australia; 2 Centre for Mental Health, The University of Melbourne, Melbourne, VIC, Australia; 3 School of Public Health, The University of Queensland, Brisbane, QLD, Australia; 4 Queensland Centre for Mental Health Research, Brisbane, QLD, Australia; 5 Institute for Health Metrics and Evaluation, University of Washington, Seattle, WA, USA

**Keywords:** Mental disorders, substance use disorders, prevalence, epidemiology, severity

## Abstract

**Objective:**

Mental and substance use disorders are the leading causes of disability worldwide. Contemporary estimates of prevalence, severity and impairment are essential for service planning. This study provides estimates of prevalence, severity, impairment and demographic correlates of mental and substance use disorders in 2020–22 and changes in prevalence since 2007.

**Methods:**

Data were from the two Australian National Surveys of Mental Health and Wellbeing conducted in 2020-22 (*N* = 15,893) and 2007 (*N* = 8841). Descriptive statistics report prevalence of lifetime and 12-month mental and substance use disorder by sex and age, proportion of people with each mental disorder by levels of severity (mild, moderate and severe) and mean days out of role by mental disorder class (mood, anxiety, substance use). Logistic regression analyses examined demographic correlates of mental disorder class and assessed changes over time.

**Results:**

The lifetime prevalence of any mental or substance use disorder in 2020–22 was 40.2%. The 12-month prevalence was 20.2% (mood disorder - 7.4%, anxiety disorder - 15.7% and substance use disorder - 3.1%). Mood disorders were associated with significant impairment. The prevalence of mental disorders has changed over time, with mood and anxiety disorders increasing and substance use disorders decreasing. These changes were most evident among young adults.

**Conclusion:**

Mental disorders are common in Australia. Impairment associated with mental disorders remains significant. Particular focus should be paid to young adults aged 16–25 years who have shown the largest increases in anxiety and mood disorder prevalence over the past 13 years.

## Introduction

Quantifying and characterising mental and substance use disorders in the general population are vital both for understanding their prevalence and distribution as well as developing prevention, treatment and policy responses to these population health challenges. Indeed, accurate estimates of prevalence and severity have led to the recognition that mental and substance use disorders are the leading cause of disability worldwide (Global Burden of Disease Study, 2015). Published evidence of shifts in the mental health of the Australian population over the last 15–20 years come from studies using broad mental health screening scales, such as the Kessler Psychological Distress Scale and the Mental Health Index 5 (Botha et al., 2023, Slade et al., 2024). These studies show increases in the prevalence of mental health problems over time, particularly among young adults. However, population level changes in the prevalence of mental and substance use disorders, ascertained through detailed diagnostic interviews, are currently unknown.

In Australia, there have been two previous general population surveys estimating the prevalence of mental and substance use disorders. In 1997, results from the first Australian National Survey of Mental Health and Wellbeing (NSMHWB) demonstrated that one in five Australians met criteria for any mental or substance use disorder in the past 12 months, that mental and substance use disorders were associated with significant disability and impairment (Andrews et al., 2001), that few people sought help for mental disorders (Parslow and Jorm, 2000) and that many people experienced more than one mental disorder (Andrews et al., 2002). The second NSMHWB was carried out in 2007. While there were some critical differences in methodology between the 2007 and the 1997 survey, the results demonstrated that, once again, around one in five Australians met criteria for any mental or substance use disorder (Slade et al., 2009), that rates of service use among those with mental disorders were less than optimal (Burgess et al., 2009) and that comorbidity was common (Teesson et al., 2009).

A third national survey of mental and substance use disorders was carried out between 2020 and 2022. A priority of this third survey was to employ methodology and instrumentation that allowed for a direct comparison with the 2007 NSMHWB. A further priority was to report not just the prevalence of mental disorders but also to describe the socio-demographic correlates, severity and impairment associated with mental disorders. The aims of this paper are to present updated estimates of the prevalence, socio-demographic correlates, severity and impairment of mental and substance use disorders and examine any changes in prevalence over time.

## Methods

### Sample

This study presents findings from the 2020–22 National Study of Mental Health and Wellbeing (NSMHWB), a component of the wider Intergenerational Health and Mental Health Study funded by the Australian Government Department of Health and Aged Care and conducted by the Australian Bureau of Statistics (ABS). The sample represents all usual residents in Australian aged 16–85 years living in private dwellings in urban and rural areas across all states and territories. Very remote parts of Australia, including discrete Aboriginal and Torres Strait Islander communities were not included. The 2020–22 NSMHWB involved two separate cohorts of respondents, the first cohort was interviewed between December 2020 and July 2021 (*N* = 5554) and the second between December 2021 and October 2022 (*N* = 10,339). This paper presents findings based on both cohorts combined (*N* = 15,893). Given this is a secondary analysis of publicly available data, this study was exempt from human ethics review. Using a stratified area sample of private dwellings, households were randomly selected and one person aged 16–85 years was randomly selected to complete the survey. Those aged 16–24 years were over-sampled (i.e. had a higher probability of selection) to improve estimates in this age group. Further methodological detail is provided elsewhere (ABS, 2020–2022). There were 15,893 fully responding households, representing a response rate of 52%. Among the 48% who did not respond, 10% refused, 20.5% were unable to be contacted, and 17.5% provided partial responses only. The achieved sample of 15,893 represents an estimated population count of 19,828,348 Australian adults. The total sample counts (with associated unweighted percentages) and estimated population counts (with associated weighted percentages), categorised by various socio-demographic variables, are displayed in Table 1.

**Table 1.** Survey respondent characteristics.

	Sample count	Unweighted (%)	EPC (’000)	Weighted (%)
Sex at birth^*^				
Male	7409	46.6	9762.8	49.2
Female	8482	53.4	10,065.6	50.8
Age (years)				
16–25	1619	10.2	2707.7	13.7
26–45	5399	34	7133.7	36
46–65	4560	28.7	6188.4	31.2
66–85	4313	27.1	3794.9	19.1
Marital status				
Married	7267	45.7	10179.2	51.3
Widowed/separated/divorced	3445	21.7	2830.5	14.3
Never married	5179	32.6	6815.2	34.4
Labour force status				
Employed	10,275	64.7	12,969.8	65.4
Unemployed	325	2	627.4	3.2
Not in the labour force	5291	33.3	6227.6	31.4
Education				
Post-school qualification	10,674	67.2	11,495.6	58
School qualification only	2061	13	3451.1	17.4
Did not complete school	2910	18.3	4352.8	22
Unknown	246	1.5	525.3	2.6
Country of birth				
Overseas	5250	33	6611.2	33.3
Australia	10,641	67	13213.6	66.7
Remoteness area				
Major cities	12,038	75.8	14,803.0	74.7
Inner regional	2626	16.5	3350.6	16.9
Outer regional or remote	1227	7.7	1671.3	8.4
SEIFA (Index of relative socio-economic disadvantage, quintiles)				
1 (Most disadvantaged)	2688	16.9	3495.2	17.6
2	2748	17.3	3461.6	17.5
3	3051	19.2	3689.0	18.6
4	3969	25	4882.5	24.6
5 (Most advantaged)	3435	21.6	4296.5	21.7
Total	15,891		19,824.8	

EPC: estimated population count; NSMHWB: National Study of Mental Health and Wellbeing.

* Total excludes persons who described their sex at birth as Another term.

### Weights

Weights were computed by the ABS based on the inverse probability of selection. These weights were calibrated to align with the estimated resident Australian population as of March 2021. However, since the sample may have underrepresented certain demographic variables, such as educational attainment, household composition, and labour force status, the weights were further adjusted using benchmarks obtained from the ABS monthly Labour Force Survey conducted from December 2020 to July 2021.

### Survey interview

The 2020–22 NSMHWB, like the 2007 NSMWHB, collected information on a broad range of mental and substance use disorders as well as health risk factors (e.g. smoking), physical health, severity, impairment, functioning, disability, societal participation, service use, and perceived need for care. The content of the interview was developed in consultation with a reference group that consisted of academic experts, people with lived experience, carers and government representatives as well as survey methodologists from the ABS. The survey development process included cognitive testing, pilot testing and dress rehearsal phases.

### Interview procedure

The 2020–22 NSMHWB was conducted by interviewers from the ABS who had prior interviewing experience. In addition, all interviewers underwent a 4-day training programme that emphasised survey concepts and definitions. Face-to-face interviews were conducted in the respondent’s household using a computer-assisted personal interview (CAPI) questionnaire. Due to the sensitive and personal nature of some content, interviews were conducted in private, and participation was voluntary. Participation was not remunerated.

### Diagnostic assessment

Diagnostic information was collected using the World Health Organization Composite International Diagnostic Interview, version 3.0 (WHO-CIDI) (Kessler and Ustün, 2004), according to the definitions and criteria outlined in the Diagnostic and Statistical Manual of Mental Disorders, Fourth Edition (*DSM*-IV). The WMH-CIDI has been used extensively to across the globe as part of the World Mental Health Survey Initiative (Scott et al., 2018). The WMH-CIDI uses a lifetime time frame. Questions on experiences of symptoms in the previous 12 months were combined with lifetime diagnoses to establish 12-month diagnoses. Diagnostic exclusion rules were applied, unless explicitly stated. The mental and substance use disorders covered in the 2020–22 NSMHWB were the same as those in the 2007 survey, and categorised into three disorder classes: mood disorders, anxiety disorders, and substance use disorders. Mood disorders included major depressive disorder, dysthymia and bipolar disorder. Anxiety disorders included agoraphobia (with or without panic disorder), social phobia (now known as social anxiety disorder), panic disorder, generalised anxiety disorder (GAD), obsessive-compulsive disorder (OCD), and post-traumatic stress disorder (PTSD). Substance use disorders included abuse and dependence for alcohol, cannabis, sedatives, stimulants, and opioids. Note that this paper focusses on the prevalence of individual mental and substance use disorders. See Sunderland et al. (in press) for more detail on mental and substance use disorder comorbidity.

### Socio-demographic characteristics

The demographic section included questions related to age, sex at birth, gender, sexual orientation, country of birth, main language spoken and marital status, Indigenous status, labour force status, educational attainment and personal income. To conform with the latest ABS standards and commonly used ABS questions from other surveys, some of the socio-demographic questions were updated from the 2007 NSMHWB.

### Impairment, severity, disability and functioning

The Sheehan Disability Scale assessed the impairment caused by a mental disorder in four domains of life: home management, ability to work, ability to form and maintain close relationships with other people, and social life (Buist-Bouwman et al., 2008). Participants rated the degree of impairment separately for each mental disorder on a scale of 1–10 for each domain. The scores for each domain were then categorised into: mild (1–3), moderate (4–6) and severe (7–10). A severity measure was derived to assess the impact of 12-month mental disorders. A person was considered to have a severe disorder if they met diagnostic criteria for bipolar disorder, or experienced a suicide attempt, or met criteria for a mental or substance use disorder and experienced at least two areas of severe impairment on the Sheehan Disability Scale associated with that disorder. Moderate disorder was defined as meeting criteria for a mental or substance use disorder and experiencing moderate (but not severe) impairment in any area on the Sheehan Disability Scale associated with that disorder. Mild disorder was defined as meeting criteria for a mental or substance use disorder but experiencing neither moderate nor severe impairment on the Sheehan Disability Scale associated with that disorder.

The Kessler 10 (K10; [Kessler et al., 2003]) was used to assess psychological distress in the 4 weeks prior to interview (analyses of the K10 are not reported in this paper). Impairment was assessed with a composite of two questions at the end of the K10 scale asking about the impact of ‘these feelings’ (i.e. the feelings asked about in the K10 scale) on the ability to carry out usual daily activities. Respondents were asked how many days in the past 4 weeks they were totally unable to carry out their normal activities and how many days they had to cut down on their normal activities as a result of these feelings. As was done in the 2007 NSMHWB, overall ‘days out of role’ was calculated by summing days totally unable to carry out normal activities and 0.6 times days cut-back.

### Data analysis

All analyses were performed using R Statistical Software (R, 2024) within the ABS DataLab environment. Summary statistics were calculated for survey respondent characteristics and presented as weighted and unweighted percentages. Descriptive tables display the lifetime prevalence, 12-month prevalence, and severity of mental disorders, with modified Wilson 95% confidence intervals (Korn and Graubard, 1998). Lifetime prevalence was stratified by sex at birth, and 12-month prevalence was stratified by sex at birth and age (categorised into 16–25, 26–45, 46–65 and 66+ years). The mean (and standard error of the mean) days out of role were calculated by diagnostic category and sex at birth. Independent samples *t*-tests were used to compare days out of role between males and females. Logistic regression models were used to examine the socio-demographic correlates of 12-month mood, anxiety, substance use and any mental disorders.

Changes over time were tested in a data set combining both the 2020–22 and 2007 survey data (Moser et al., 2013). To measure the difference in prevalence between the two surveys, logistic regression models were specified, regressing each individual mental disorder on survey year (coded as 0 = 2007 survey and 1 = 2020–22 survey). Models were run for all respondents and then repeated to explore whether changes over time were different for different sex and age groups. This was achieved by running a model that included a sex-by-survey year interaction term and a separate model that included an age-by-survey year interaction term. Poisson regression models were used to investigate changes over time in days out of role by diagnostic category. Proportional odds logistic regression was used to examine changes over time in severity, with odds ratios derived from these models representing the average odds of being one severity step higher (i.e. moderate vs mild or severe vs moderate). All weighted calculations were made using replicate weights provided by the ABS and were computed using the survey R package.

## Results

### Prevalence

In the 2020–22 NSMHWB, two out of every five Australian adults, 40.2%, 95% confidence interval (CI) = 39.2–41.3, met criteria for a *DSM*-IV mental or substance use disorder at some point in their lifetime (Table 2). Anxiety disorders (24.8%, 95% CI = 24.0–25.6) were the most common disorder class, followed by substance use disorders (18.8%, 95% CI = 18.1–19.6) and then mood disorders (15.3%, 95% CI = 14.5–16.2). While the lifetime prevalence of any disorder did not differ between males and females, females had a higher prevalence of mood and anxiety disorders than males (mood disorder: 18.2%, 95% CI = 16.9–19.6 in females vs 12.4%, 95% CI = 11.4–13.5 in males; anxiety disorder: 29.7%, 95% CI = 28.3–31.1 in females vs 19.7%, 95% CI = 18.6–20.9 in males) and males had a higher prevalence of substance use disorders than females (25.1%, 95% CI = 23.8–26.5 in males vs 12.7%, 95% CI = 11.9–13.6 in females).

**Table 2.** Prevalence of lifetime mental disorders, by sex.

	Total sample			Males			Females		
	EPC (‘000)	%	95% CI	EPC (‘000)	%	95% CI	EPC (‘000)	%	95% CI
Any mood (affective) disorder	3041.7	15.3	(14.5–16.2)	1210.0	12.4	(11.4–13.5)	1828.7	18.2	(16.9–19.6)
Any anxiety disorder	4913.0	24.8	(24.0–25.6)	1923.3	19.7	(18.6–20.9)	2986.2	29.7	(28.3–31.1)
Any substance use disorder	3731.5	18.8	(18.1–19.6)	2452.0	25.1	(23.8–26.5)	1279.5	12.7	(11.9–13.6)
Any mental disorder	7976.3	40.2	(39.2–41.3)	3925.7	40.2	(38.8–41.6)	4047.1	40.2	(38.7–41.7)

EPC: estimated population count; 95% CI: 95% confidence interval.

The prevalence of any 12-month mental or substance use disorder was 20.2% (95% CI = 19.5–21.0, Table 3). In contrast to lifetime prevalence, 12-month prevalence of any mental or substance use disorder was higher in females (23.1%, 95% CI = 22.0–24.2) than males (17.3%, 95% CI = 16.3–18.4). Females had a higher prevalence of any 12-month mood disorder (females: 8.7%, 95% CI = 8.0–9.6; males: 6.1%, 95% CI = 5.4–6.8) and anxiety disorder (females: 19.3%, 95% CI = 18.3–20.5; males: 12.0%, 95% CI = 11.1–13.0). The higher prevalence of 12-month anxiety disorders in females was particularly driven by panic disorder, agoraphobia and PTSD. Males had a higher prevalence of 12-month substance use disorders (4.2%, 95% CI = 3.7–4.9) than females (2.1%, 95% CI = 1.8–2.5). For all disorder classes, the prevalence was highest in the 16–25 age group and generally decreased as age increased (see Table 3).

**Table 3.** Prevalence of individual 12-month *DSM*-IV mental disorders, by sex and age.

	Total		Males		Females		Aged 16-25		Aged 26-45		Aged 46-65		Aged 66 +	
	%	95% CI	%	95% CI	%	95% CI	%	95% CI	%	95% CI	%	95% CI	%	95% CI
Mood disorders														
Major depressive disorder	6.3	(5.9–6.8)	5.3	(4.7–6)	7.3	(6.7–8)	10.5	(9–12.2)	6.8	(6–7.8)	5.9	(5.1–6.9)	2.5	(2–3.1)
Dysthymia	1.6	(1.4–1.9)	1.5	(1.2–1.9)	1.8	(1.5–2.1)	1.9	(1.3–2.8)	1.8	(1.4–2.4)	1.6	(1.2–2.1)	1.1	(0.7–1.7)
Bipolar disorder	0.9	(0.7–1.2)	0.6	(0.4–0.9)	1.2	(0.9–1.7)	2.0	(1.4–2.9)	1.1	(0.7–1.5)	NA		NA	
*Any mood disorder*	*7.4*	*(6.9–7.9)*	*6.1*	*(5.4–6.8)*	*8.7*	*(8–9.6)*	*12.8*	*(11.3–14.4)*	*8.0*	*(7.1–9)*	*6.8*	*(5.9–7.7)*	*2.8*	*(2.3–3.5)*
Anxiety disorders														
Generalised anxiety disorder	3.5	(3.1–3.9)	2.5	(2.1–3.1)	4.4	(3.9–5)	5.6	(4.7–6.8)	4.4	(3.7–5.3)	2.5	(1.9–3.2)	1.4	(1–1.9)
Panic disorder	2.7	(2.4–3.1)	1.7	(1.4–2.2)	3.7	(3.1–4.3)	6.0	(4.8–7.6)	2.9	(2.4–3.5)	1.9	(1.4–2.5)	0.9	(0.6–1.4)
Agoraphobia with/without panic disorder	2.2	(1.9–2.6)	1.4	(1.1–1.9)	3.0	(2.5–3.6)	4.7	(3.7–5.9)	2.1	(1.6–2.6)	2.0	(1.6–2.6)	0.8	(0.5–1.3)
Social anxiety disorder	7.7	(7.2–8.4)	6.0	(5.3–6.8)	9.4	(8.7–10.2)	17.2	(15.3–19.2)	8.7	(7.8–9.6)	5.1	(4.2–6.1)	2.4	(1.9–3.1)
Obsessive-compulsive disorder	4.2	(3.9–4.6)	3.4	(2.8–4.1)	5.0	(4.4–5.7)	8.2	(7–9.5)	4.8	(4.1–5.6)	2.8	(2.2–3.6)	2.0	(1.5–2.8)
Post-traumatic stress disorder	4.0	(3.6–4.5)	2.4	(2–2.9)	5.6	(5–6.2)	6.3	(5–7.9)	4.3	(3.7–5)	3.8	(3.1–4.6)	1.9	(1.5–2.4)
*Any anxiety disorder*	*15.7*	*(15–16.5)*	*12.0*	*(11.1–13)*	*19.3*	*(18.3–20.5)*	*28.4*	*(26.1–30.8)*	*17.5*	*(16.3–18.7)*	*12.5*	*(11.2–13.9)*	*7.1*	*(6–8.3)*
Substance use disorders														
Alcohol use disorder	2.5	(2.2–2.8)	3.2	(2.7–3.8)	1.8	(1.5–2.1)	5.7	(4.4–7.3)	2.8	(2.2–3.4)	1.5	(1.1–2.1)	0.8	(0.5–1.3)
Cannabis use disorder	0.6	(0.5–0.9)	1.0	(0.7–1.4)	0.3	(0.2–0.5)	2.5	(1.7–3.6)	0.5	(0.4–0.8)	NA		NA	
Other substance use disorder	0.3	(0.2–0.5)	0.4	(0.3–0.7)	0.2	(0.1–0.4)	0.6	(0.3–1.2)	0.6	(0.4–0.9)	NA		NA	
*Any substance use disorder*	*3.1*	*(2.8–3.5)*	*4.2*	*(3.7–4.9)*	*2.1*	*(1.8–2.5)*	*7.9*	*(6.5–9.7)*	*3.4*	*(2.8–4.2)*	*1.7*	*(1.3–2.3)*	*0.9*	*(0.6–1.4)*
***Any mental disorder***	***20.2***	***(19.5–21)***	***17.3***	***(16.3–18.4)***	***23.1***	***(22–24.2)***	***35.5***	***(32.9–38.1)***	***22.1***	***(20.6–23.7)***	***16.8***	***(15.3–18.5)***	***9.3***	***(8.3–10.5)***

95% CI: 95% confidence interval; Other substance use disorder: any Stimulant, Sedative, or Opioid use disorder; NA: Not available.

### Socio-demographic correlates

Results from multivariable logistic regression models examining the association between 12-month mental and substance use disorder classes and socio-demographic characteristics are shown in Table 4. Age was uniquely and monotonically associated with all mental and substance use disorder classes with the highest odds of any 12-month mental or substance use disorder found in the 16–25 years compared to the 66–85 years age group (odds ratio [OR] = 4.24, 95% CI = 3.33–5.41). Marital status was uniquely associated with all mental and substance use disorder classes and any mental or substance use disorder, with those who were separated, widowed, divorced or never married having a higher odds of any mental or substance use disorder compared to those who were married (Separated/widowed/divorced: OR = 1.88, 96% CI = 1.58–2.24; Never married; OR = 1.73, 95% CI = 1.50–2.00). Compared to being employed, being unemployed or not in the labour force was associated with a higher odds of any mood disorder (Unemployed: OR = 2.00, 95% CI = 1.30–3.09; Not in the labour force: OR = 1.73, 95% CI = 1.41–2.12) and any anxiety disorder (Unemployed: OR = 1.72, 95% CI = 1.29–2.30; Not in the labour force: OR = 1.37, 95% CI = 1.17–1.61), yet was unrelated to any substance use disorder. The odds of all mental disorder classes were lower among those born in Australia compared to those born overseas (OR = 0.56, 95% CI = 0.48–0.65 for any mental or substance use disorder). There was little evidence of association between mental disorder classes and education status, remoteness and socioeconomic disadvantage.

**Table 4.** Socio-demographic correlates of people with 12-month *DSM*-IV mental disorders.

	Any mood disorder		Any anxiety disorder		Any substance use disorder		Any mental disorder	
	aOR^a^	95% CI	aOR^a^	95% CI	aOR^a^	95% CI	aOR^a^	95% CI
Age at survey (years)								
16–25	4.81	(3.08–7.52)	4.34	(3.28–5.74)	3.76	(1.86–7.60)	4.24	(3.33–5.41)
26–45	4.35	(2.96–6.40)	3.18	(2.41–4.19)	2.58	(1.24–5.38)	3.11	(2.45–3.96)
46–65	3.48	(2.58–4.70)	2.21	(1.68–2.90)	1.56	(0.81–3.00)	2.29	(1.84–2.86)
66–85	1.00	(1.00–1.00)	1.00	(1.00–1.00)	1.00	(1.00–1.00)	1.00	(1.00–1.00)
*Wald Chi*^2^ *(p value)*	*74.54*	*<0.001*	*117.34*	*<0.001*	*22.81*	*<0.001*	*151.38*	*<0.001*
Sex at birth								
Male	1.00	(1.00–1.00)	1.00	(1.00–1.00)	1.00	(1.00–1.00)	1.00	(1.00–1.00)
Female	1.36	(1.13–1.64)	1.74	(1.52–2.00)	0.48	(0.38–0.61)	1.40	(1.25–1.57)
*Wald Chi*^2^ *(p value)*	*11.67*	*0.001*	*65.98*	*<0.001*	*41.7*	*<0.001*	*35.04*	*<0.001*
Marital status								
Married	1.00	(1.00–1.00)	1.00	(1.00–1.00)	1.00	(1.00–1.00)	1.00	(1.00–1.00)
Separated/Divorced/Widowed	2.88	(2.24–3.72)	1.70	(1.37–2.11)	1.86	(1.12–3.11)	1.88	(1.58–2.24)
Never married	1.87	(1.47–2.37)	1.62	(1.39–1.88)	2.53	(1.72–3.73)	1.73	(1.50–2.00)
*Wald Chi*^2^ *(p value)*	*77.21*	*<0.001*	*49.41*	*<0.001*	*23.54*	*<0.001*	*77.19*	*<0.001*
Labour Force Status								
Employed	1.00	(1.00–1.00)	1.00	(1.00–1.00)	1.00	(1.00–1.00)	1.00	(1.00–1.00)
Unemployed	2.00	(1.30–3.09)	1.72	(1.29–2.30)	1.65	(0.90–3.04)	1.73	(1.29–2.32)
Not in the Labour Force	1.73	(1.41–2.12)	1.37	(1.17–1.61)	0.82	(0.52–1.27)	1.36	(1.18–1.56)
*Wald Chi*^2^ *(p value)*	*31.6*	*<0.001*	*27.95*	*<0.001*	*4.7*	*0.095*	*25.99*	*<0.001*
Education								
Post-school qualification	1.00	(1.00–1.00)	1.00	(1.00–1.00)	1.00	(1.00–1.00)	1.00	(1.00–1.00)
School qualification only	0.96	(0.73–1.25)	0.91	(0.77–1.08)	1.09	(0.77–1.54)	0.93	(0.80–1.08)
Did not complete school	0.79	(0.59–1.04)	0.82	(0.66–1.01)	0.91	(0.61–1.36)	0.82	(0.67–0.99)
*Wald Chi*^2^ *(p value)*	*3.02*	*0.221*	*4.61*	*0.1*	*0.54*	*0.763*	*4.83*	*0.089*
Country of birth								
Australia	0.62	(0.50–0.77)	0.58	(0.49–0.69)	0.47	(0.33–0.67)	0.56	(0.48–0.65)
Overseas	1.00	(1.00–1.00)	1.00	(1.00–1.00)	1.00	(1.00–1.00)	1.00	(1.00–1.00)
*Wald Chi*^2^ *(p value)*	*20.67*	*<0.001*	*42.5*	*<0.001*	*18.76*	*<0.001*	*62.25*	*<0.001*
Remoteness area								
Major cities	1.00	(1.00–1.00)	1.00	(1.00–1.00)	1.00	(1.00–1.00)	1.00	(1.00–1.00)
Inner regional	1.09	(0.89–1.35)	1.08	(0.89–1.31)	1.17	(0.81–1.70)	1.04	(0.88–1.23)
Outer regional or remote	0.99	(0.65–1.51)	0.93	(0.72–1.19)	1.27	(0.74–2.19)	0.96	(0.75–1.22)
*Wald Chi*^2^ *(p value)*	*0.77*	*0.681*	*0.89*	*0.642*	*1.25*	*0.535*	*0.32*	*0.851*
SEIFA (Index of Relative Socioeconomic Disadvantage)								
1 (Most disadvantaged)	0.85	(0.66–1.08)	0.99	(0.82–1.20)	0.79	(0.51–1.23)	0.96	(0.82–1.12)
2	1.03	(0.75–1.41)	1.12	(0.93–1.34)	1.04	(0.69–1.58)	1.06	(0.90–1.26)
3	0.85	(0.62–1.15)	1.00	(0.82–1.21)	1.15	(0.74–1.78)	1.01	(0.85–1.19)
4	1.00	(0.74–1.35)	0.98	(0.82–1.16)	1.05	(0.74–1.50)	0.98	(0.83–1.15)
5 (Most advantaged)	1.00	(1.00–1.00)	1.00	(1.00–1.00)	1.00	(1.00–1.00)	1.00	(1.00–1.00)
*Wald Chi*^2^ *(p value)*	*4.89*	*0.298*	*2.81*	*0.59*	*3.43*	*0.488*	*1.47*	*0.832*

95% CI: 95% confidence interval.

a Adjusted odds ratio. In addition to the correlates listed in the table, all models adjust for sexuality and Aboriginal and Torres Strait Islander status.

#### Severity

Among those who met criteria for each 12-month mental disorder class, the proportions who were classified as mild, moderate or severe are shown in Table 5. About one quarter of people who met criteria for any 12-month mental disorder were classified as mild (27.6%, 95% CI = 25.5–29.8), around half as moderate (46.6%, 95% CI = 45.0–48.3) and a quarter as severe (25.7%, 95% CI = 23.8–27.6). When comparing disorder classes, people with a 12-month mood disorder were more often classified as severe (46.4%) compared to people with a 12-month anxiety disorder (27.2%) or a 12-month substance use disorder (25.3%). Moderate severity was evident in a greater proportion of people with 12-month mood disorders (46.2%) and people with 12-month anxiety disorders (47.0%) compared to people with 12-month substance use disorders (32.2%).

**Table 5.** Severity of 12-month *DSM*-IV mental disorders.

	Mild		Moderate		Severe	
	%	95% CI	%	95% CI	%	95% CI
Any mood disorder	7.5	(5.5–10.1)	46.2	(42.2–50.2)	46.4	(42.6–50.2)
Any anxiety disorder	25.6	(23.5–27.8)	47.0	(45.1–49)	27.2	(25.2–29.3)
Any substance use disorder	42.5	(36.8–48.4)	32.2	(26.7–38.3)	25.3	(21–30.2)
Any mental disorder	27.6	(25.5–29.8)	46.6	(45–48.3)	25.7	(23.8–27.6)

95% CI: 95% confidence interval.

### Impairment

Those who met 12-month criteria for any mental disorder reported, on average, around 4 days out of role in the 28 days prior to the survey (see Table 6). Days out of role were greatest for those with any mood disorder (Mean = 6.51, SEM = 0.32), followed by those with any anxiety disorder (Mean = 4.15, SEM = 0.17) and then any substance use disorder (Mean = 3.89, SEM = 0.40). Females with any anxiety disorder reported significantly more days out of role (Mean = 4.45, SEM = 0.24) than males with any anxiety disorder (Mean = 3.68, SEM = 0.25; *t* = 2.11, *p* = 0.039). Similarly, females with any substance use disorder reported significantly more days out of role (Mean = 5.32, SEM = 0.61) than males with any substance use disorder (Mean = 3.16, SEM = 0.53; *t* = 2.28, *p* = 0.011). There were no sex differences in the mean number of days out of role reported by people with a mood disorder.

**Table 6.** Days out of role in the last 28 days, by 12-month *DSM*-IV mental disorder class.

	Total sample		Males		Females		*t*-test for difference between males and females	
	Mean	SEM	Mean	SEM	Mean	SEM	*t*	*p* value
Any mood disorder	6.51	0.32	6.49	0.52	6.53	0.48	0.05	0.961
Any anxiety disorder	4.15	0.17	3.68	0.25	4.45	0.24	2.11	0.039
Any substance use disorder	3.89	0.40	3.16	0.53	5.32	0.61	2.62	0.011
Any mental disorder	4.09	0.17	3.63	0.23	4.43	0.24	2.28	0.026
No mental disorder	0.79	0.04	0.71	0.04	0.88	0.05	2.90	0.005

SEM: Standard error of the mean.

### Changes in prevalence, severity and impairment between 2007 and 2020–22

Changes in prevalence between the 2007 and 2020–22 surveys are shown in Table 7 as odds ratios representing the odds of meeting criteria for each 12-month disorder class in the 2020–22 survey compared to the 2007 survey. The odds of meeting criteria for any 12-month mood disorder are 1.18 times higher (95% CI = 1.08–1.29) in the most recent survey than in the earlier survey. This overall increase is driven by an increased odds of meeting criteria for any anxiety disorder (OR = 1.40, 95% CI = 1.26–1.55) and any mood disorder (OR = 1.30, 95% CI = 1.12–1.5). In contrast, the odds of any substance use disorder are lower in the most recent compared to the previous survey (OR = 0.61, 95% CI = 1.08–1.29).

**Table 7.** Changes between 2007 and 2020–22 in 12-month mental disorder classes, by sex and age.

	Total		Males		Females		Aged 16–25		Aged 26–45		Aged 46–65		Aged 66–85	
	OR	95% CI	OR	95% CI	OR	95% CI	OR	95% CI	OR	95% CI	OR	95% CI	OR	95% CI
Any mood disorder	1.30	(1.12–1.5)	1.33	(1.02–1.74)	1.27	(1.06–1.52)	2.85	(2.08–3.91)	1.04	(0.82–1.33)	1.23	(0.89–1.69)	1.13	(0.72–1.78)
Any anxiety disorder	1.40	(1.26–1.55)	1.39	(1.16–1.68)	1.41	(1.22–1.62)	2.90	(2.28–3.69)	1.29	(1.11–1.5)	1.01	(0.82–1.24)	1.94	(1.35–2.78)
Any substance use disorder	0.61	(0.5–0.73)	0.59	(0.46–0.75)	0.64	(0.48–0.85)	0.65	(0.48–0.86)	0.54	(0.39–0.75)	0.87	(0.53–1.42)	1.27	(0.58–2.78)
Any mental disorder	1.18	(1.08–1.29)	1.10	(0.94–1.29)	1.25	(1.12–1.4)	1.85	(1.53–2.24)	1.04	(0.91–1.2)	1.10	(0.89–1.36)	1.59	(1.2–2.12)

OR: odds ratio; 95% CI: 95% confidence interval.

Interactions between survey year (2020–22 vs 2007) and sex tested whether changes over time in prevalence differed for males and females. Results demonstrated little evidence of sex difference in the change in prevalence of mood, anxiety and substance use disorders over time. Interactions with age revealed that increases over time in the prevalence of mood disorders were driven by increases only in the 16–25 years age group. Increases in anxiety disorders were evident in all age groups except the 46–65 years age group. Decreases in the prevalence of any substance use disorder were apparent in those aged 16–45 years.

Overall, the severity of any mental or substance use disorder increased over the two surveys with the odds of being in a higher vs lower severity category (i.e. severe vs moderate or moderate vs mild) approximately 1.6 times higher in the 2020–22 survey compared to the 2007 survey (OR = 1.59, 95% CI = 1.34–1.89). This increase was driven mostly by an increase in the severity of substance use disorders (OR = 1.61, 95% CI = 1.15–2.24) and somewhat by an increase in the severity of anxiety disorders (OR = 1.35, 95% CI = 1.1–1.65). There is little evidence of a change in the severity of mood disorders over time (OR = 1.13, 95% CI = 0.83–1.52). Similarly, changes over time in the number of days out of role for 12-month mental disorder classes indicate that substance use disorders are associated with more days out of role in the 2020–22 survey compared to the 2007 survey, relative risk (RR) = 1.43, 95% CI = 1.01–2.02. Impairment, as measured by days out of role, associated with mood and anxiety disorders did not change between the two surveys.

## Discussion

### Summary of findings

The findings from the 2020-22 NSMHWB highlight the persistently high prevalence of mental and substance use disorders within the Australian adult population. Approximately two out of every 5 (40.2%) adults experienced at least one *DSM*-IV mental or substance use disorder in their lifetime and one out of every 5 (20.2%) experienced a mental or substance use disorder in the past 12 months. Despite development of, and investment in, evidence-based treatment of and policy responses for mental and substance use disorders in Australia, the prevalence of these disorders remains high and, in some cases, is increasing.

Anxiety disorders were the most prevalent class of 12-month disorder, followed by substance use and mood disorders. However, severity was particularly marked for mood disorders, with over 45% of those experiencing them classified as severe. In addition, mental disorders were associated with significant impairment on daily life, averaging 4 days a month being unable to carry out or having to cut back on normal daily life activities. These findings also highlight important associations between mental disorders and socio-demographic characteristics. Most notably, the 12-month prevalence of all mental disorder classes was highest for those aged 16–25 years at the time of interview.

### Comparison with the 2007 National Survey of Mental Health and Wellbeing

The 2007 and 2020-22 NSMHWB surveys are comparable in their design, with both using the WMH-CIDI diagnostic interview, allowing direct comparison of the past 12-month prevalence of common mental disorders. Comparison with the 2007 survey reveals some important shifts in the prevalence and severity of mental health disorders over the last decade. In particular, there has been an increase in the prevalence of mood and anxiety disorders. This trend was mostly, but not exclusively, driven by increases in among young people aged 16–25 years. This finding is in line with previous Australian (Botha et al., 2023; Burns et al., 2020; Butterworth et al., 2020; Harvey et al., 2017) research and highlights a dramatic generational shift in the mental health of the Australian population. The reasons behind this shift are likely to be complex and multifactorial and related to concurrent rises in important adolescent health risk factors such as exposure to social media, physical inactivity, poor diet, sleep problems and the COVID-19 pandemic, among others. The absence of a survey year by sex interaction is noteworthy and suggests that increases in the prevalence of anxiety and mood disorders have occurred proportionally for males and females.

The 2020–22 NSMWHB also demonstrated that the prevalence of substance use disorders has declined over time. Again, this is in line with recent Australian (Livingston et al., 2016) and international (Smith et al., 2024) research, albeit focused more on alcohol and drug *use* rather than disorder. While a reduction in the prevalence of substance use disorders over time is positive there is evidence that those in the 2020–22 survey who meet criteria for a substance use disorder may be experiencing greater severity compared to those who met criteria in the 2007 survey. This suggests that while fewer individuals may be engaging in harmful substance use, those who do are likely experiencing more profound and debilitating effects, requiring a renewed investment in treatment.

### Comparison with recent international surveys

Many of these findings are congruent with other recent nationally representative surveys from around the world, some of which are part of the World Mental Health Survey Initiative, and using the WMH-CIDI, allowing for cross-country comparisons. The most comparable survey is the 2020–22 Netherlands Mental Health Survey and Incidence Study (NEMESIS-3) study, which found that anxiety disorders are the most common lifetime disorder (28.6%) followed by mood (27.6%) and substance use disorders (16.7%) (Ten Have et al., 2023). However, rates of lifetime mental disorder were higher than those in Australia, with 48% of people in the Netherlands reporting lifetime disorder compared to 40% in Australia. Following the same trends as Australia, when comparing to the 2007–2009 NEMESIS-2 survey, there is a significant increase in the 12-month prevalence of *DSM*-IV mood disorders (increasing from 6% in NEMESIS-2% to 10.8% in NEMESIS-3) and *DSM*-IV anxiety disorders (increasing from 10.1% in NEMESIS-2% to 15.6% in NEMESIS-3). However, contrary to Australia, the Netherlands report a non-significant increase in the prevalence of *DSM*-IV substance use disorders (5.5%–7.1%). The association of lower age with higher 12-month prevalence rates of mental disorders found in NEMESIS-3 echoes findings in Australia, emphasising the vulnerability of younger populations globally. This vulnerability is also emphasised by both studies revealing that increases in prevalence of mood and anxiety disorders over the last decade are largely being driven by younger cohorts.

### Strengths and limitations

This study has many strengths. First, the use of a large sample that is nationally representative of the Australian population, allows broad generalisability of our findings. Moreover, the use of the WMH-CIDI 3.0 to measure mental disorders allows for comparison of these findings with the 2007 NSMHWB and with other nationally representative mental health surveys, significantly improving our ability to investigate and interpret changes in the prevalence and distribution of mental disorders over time.

These findings should be considered in light of certain limitations. Comparability between the 2007 and 2020–2022 surveys was achieved at the expense of using the most up-to-date classification of mental disorders (i.e. *DSM*-5). There have been considerable changes in diagnostic criteria for some disorders between *DSM*-IV and *DSM*-5 (most notably PTSD and substance use disorders), thus limiting the comparability of the 2020–2022 data with international surveys based on *DSM*-5 diagnostic criteria. The response rate for the 2020–22 survey was 52%, which is lower than the 2007 survey (60%). Low response rates may impact the validity of prevalence and risk estimates through potential selective participation by socio-demographic subgroups, or by people with interest in the outcomes of interest (Wright et al., 2023). Another limitation of this study is the division of data collection into two cohorts due to COVID-19-related lockdowns, which raises potential uncertainties about the validity of combining cohorts across these periods and the direct impact of the pandemic on the observed mental health trends. The ABS report minimal differences between the two cohorts in estimates of the prevalence of mental disorders. For example, the prevalence of any *DSM*-IV mental disorder was 20.9% in cohort 1 and 19.7% in cohort 2. However, without detailed pre-pandemic trajectory modelling, it remains challenging to determine whether the changes noted are continuations of existing trends or disruptions caused by the pandemic. Further research using longitudinal data and sophisticated modelling is needed to tease apart the precise effects of COVID-19 (or other shocks) on mental health trajectories. It is noteworthy that the prevalence of mental disorders did not vary substantially by respondent state of residence at the time of the survey, despite substantial differences between states in the length and severity of pandemic -related lockdown periods during 2020 and 2021. For example, data published on the ABS website show that the prevalence of any ICD-10 12-month mental disorder in Victoria, the state with arguably the strictest and longest lockdown, was 21.4%, virtually equivalent to the national average of 21.5% (ABS, 2020–2022). While these numbers refer to ICD-10 defined mental disorders, the same pattern of findings are expected for *DSM*-IV defined mental disorders. The survey interview also does not measure difficult to assess or very low prevalence disorders (e.g. personality disorders, schizophrenia). To capture these mental disorders reliably would require clinicians or other skilled interviewers, limiting the feasibility of including these mental disorders in this survey. Moreover, the NSMHWB is a household survey, which means that people in institutions (e.g. nursing homes, hospices, hostels), people in prisons, and homeless people were not surveyed.

## Conclusion

According to the latest national survey data collected in 2020–22, the prevalence of mood and anxiety disorders has increased since 2007, particularly for young adults aged 16–25 years. Well-funded and long-term universal and selective prevention approaches together with renewed early intervention, treatment and policy responses are required if we are to address the significant and in some cases growing burden associated with mental and substance use disorders.
